# Individual and community-level associations of social determinants with pituitary adenoma disparities in the US

**DOI:** 10.1007/s11060-026-05716-y

**Published:** 2026-08-05

**Authors:** Atharva Desai, Manal Malik, Charles J. Cook, David J. Fei-Zhang, Christopher Puchi, Jill N. D’Souza, Anthony M. Sheyn, Abhinav Talwar, Jorge Gutierrez, Daniel C. Chelius, Jeffrey C. Rastatter, Rishabh Sethia

**Affiliations:** 1 University of Missouri-Kansas City School of Medicine, 3901 Rainbow Boulevard, Kansas City, KS 66160 USA; 2 Tulane School of Medicine, 1430 Tulane Ave, New Orleans, LA 70112 USA; 3 Baylor College of Medicine, 6701 Fannin Street, Houston, TX 77030 USA; 4 Northwestern University Feinberg School of Medicine, 420 E Superior St, Chicago, IL 60611 USA; 5 Department of Otolaryngology-Head and Neck Surgery, Northwestern University Feinberg School of Medicine, 675 N Saint Clair, Chicago, IL 60611 USA; 6 Division of Pediatric Otolaryngology, Ann & Robert H. Lurie Children’s Hospital of Chicago, 225 East Chicago Avenue, Chicago, IL 60611 USA; 7 Division of Pediatric Otolaryngology, Department of Otolaryngology, Louisiana State University Health Sciences Center, Children’s Hospital of New Orleans, 200 Henry Clay Ave, New Orleans, LA 70118 USA; 8 Department of Otolaryngology-Head and Neck Surgery, University of Tennessee Health Science Center, 910 Madison Avenue, Memphis, TN 38163-2242 USA; 9 Department of Otolaryngology-Head and Neck Surgery, Baylor College of Medicine, 6701 Fannin Street, Houston, TX 77030 USA; 10 Department of Pediatric Otolaryngology, Nationwide Children’s Hospital, 700 Children’s Drive, Columbus, OH 43205 USA; 11 The Ohio State University College of Medicine, 1645 Neil Ave, Columbus, OH 43210 USA

**Keywords:** Skull base, Pituitary gland, Pituitary adenoma, Social determinants of health, Yost index, Race-ethnicity, Socioeconomic status

## Abstract

**Purpose:**

Through interactional, multilevel models of social determinants of health (SDoH) incorporating comprehensive census-level and individual-level SDoH-factors, to assess whether pituitary adenoma disparities are more strongly associated with community-level factors compared to individual-level ones.

**Methods:**

This retrospective study analyzed pituitary adenoma patients nationally between 2010 and 2018 using multivariate, age-adjusted regressions and cox-proportional hazards models; covariates of sex, race-ethnicity, census-level rurality-urbanicity, census-level Yost-Index score (aggregating 7 SES-measures of education, income, housing) and clinical outcomes of mortality, surgical resection, & delays-in-treatment (3 months-or-over from diagnosis).

**Results:**

Across 44,514 pituitary adenomas-patients, all-cause mortality featured markedly positive independent predictors of decreasing Yost-SES (OR, 1.43; 95% CI, 1.35–1.52; *p* < 0.001) and Male-Sex (OR, 1.24; 95% CI, 1.16–1.32; *p* < 0.001). Indicated surgery receipt showed markedly positive independent predictors of Male-Sex (OR, 1.64; 95% CI, 1.59–1.72; *p* < 0.001), Minority Race/Ethnicity (OR, 1.18; 95% CI, 1.14–1.23; *p* < 0.001), and Rurality (OR, 1.08; 95% CI, 1.01–1.16; *p* = 0.030). Having a delay-of-treatment showed markedly positive independent predictors of Female-Sex (OR, 1.24; 95% CI, 1.18–1.30; *p* < 0.001) and decreasing Yost-SES (OR, 1.14; 95% CI, 1.08–1.20; *p* < 0.001).

**Conclusions:**

Through assessing individual- and community-level social determinants in tandem, this study identified detrimental SDoH-associations with pituitary adenoma care and prognosis. Community-level Yost-Index SES and individual sex showed stronger associations with observed disparities, presenting multilevel considerations for prospective initiatives to consider.

**Supplementary Information:**

The online version contains supplementary material available at https://doi.org/10.1007/s11060-026-05716-y.

## Introduction

With advancements in diagnostic and imaging capacities, the incidence of pituitary adenomas has consequently increased over time [1]. While most follow a benign clinical course, their potential to progress into symptomatic macroadenomas has prompted numerous cohort investigations aimed at elucidating clinicodemographic variables that influence such prognostic courses [1–3]. Among them, select factors related to age, sex, tumor type and grade, age and tumor type have been identified [4, 5].

Beyond these clinical factors, non-clinical factors have been observed to affect 80–90% of overall health outcomes [6]. These factors, known as social determinants or drivers of health (SDoH), encompass differences in social, environmental, and exposure-related conditions that influence both health status and care access. Prior investigations across a range of adult and pediatric head and neck tumors have highlighted strong associations between SDoH and patient outcomes [7–11]. For pituitary adenomas in particular, socioeconomic status factors (e.g. income level, insurance status, and community job attainment levels) and racial-ethnic identity have long been observed in prior investigations [12–14]. Despite these understood relationships, the basis of these associations arise from study design shortcomings pertaining to a combination of single-region patient catchment areas, isolated or limited variable selection in modeling, area-level variables lacking specificity beyond areas as small as zip codes, and lacking considerations or representations of sociodemographic characteristics pertaining to the individual and their surrounding community (beyond race-ethnicity and socioeconomic status) in tandem. In turn, there remains a need for more comprehensive evaluations addressing such limitations across a wider consideration of SDoH-related disparities for pituitary adenoma patients in the US.

The Yost Index is a composite, continuously updated measure comprising seven differentially weighted social determinant factors. Developed by Yost et al. in 2001, this index captures a broad range of census-level socioeconomic indicators and can be integrated with individual-level sociodemographic and census-based rurality-urbanicity measures available in a specially authorized, national dataset of the National Cancer Institute’s Surveillance, Epidemiology, and End Results (SEER) database [15, 16]. Despite its demonstrated value in understanding disparities in other head and neck cancers, the Yost Index has not been fully applied to the study of pituitary adenoma care and prognostic disparities, especially within the context of other census-/community-based SDoH-variables.

Through multilevel modeling incorporating the Yost Index alongside other individual- and community-level SDoH factors, we aimed to examine how these factors interact to shape disparities in pituitary adenoma care and outcomes nationwide. We hypothesized that increasingly worse status in community-level SDoH-factors, namely Yost Index score, would more strongly influence care and prognostic outcome disparities compared to individual-level SDoH in pituitary adenoma patients.

## Materials and methods

This retrospective cohort study follows the Strengthening the Reporting of Observational Studies in Epidemiology (STROBE) reporting guideline. Per the protocols of Northwestern University’s Institutional Review Board, this study did not require prior IRB/ethics committee approval or waiver of informed consent, as the data consisted of publicly available, deidentified patient data.

### Database

The National Cancer Institute-SEER program contains national datasets of patient variables, pathological characteristics, treatment modalities, and prognostic outcomes. This study used the specially authorized Head-Neck SEER 2020 Dataset, which is comprised of 18 registries across the country representative of the variation observed within the national demographic of cancer patients in the US.

As a unique feature of this dataset, census-level SDoH measurements, including a composite socioeconomic status (SES) measure called the Yost Index, and two rurality-urbanicity measures (by Rural-Urban Commuting Area (RUCA) codes and Urban Rural Indicator Codes (URIC)) are characterized on the individual-patient level. In particular, the Yost Index comprises a differentially-weighted, aggregate measure of several SDoH-factors: proportion of residents with at least a high-school level education, median household income, percentage living below poverty, median home value, median rent, proportion working blue-collar jobs, and proportion over 16-years-old in the workforce without employment. Using these measures scores are reported on a scale from 1 to 100 with 1 being the highest SES and 100 being the lowest. These scores are then divided into quintiles labelled as “Highest”, “Higher”, “Middle”, “Lower”, and “Lowest”. As part of the dataset restrictions, these labels are already pre-assigned to each individual patient without reference to their geographic location in order to remain compliant with data privacy agreements.

Clinical measures were also present within this dataset and consisted of all-cause mortality, receipt of first-line surgery, and delay of treatment initiation (defined as 3 months or more after primary tumor diagnosis). Receipt of treatment modalities observed were all considered indicated per the SEER-categorization variable of “Reason for Indication/Non-Indication”.

### Characteristic and outcome definitions

The SEER dataset was searched for adult patients (20 + years) diagnosed with a pituitary adenoma from 2010 to 2018. The International Classification of Diseases for Oncology, 3rd Edition (ICD-O-3) was used for determining the primary site. These patients were then categorized based on a total of four clinicodemographic factors, two individual-level and two census tract-level. The two individual-level categories were biological sex and race-ethnicity (Non-Hispanic White, Hispanic, Asian or Pacific Islander, Black, Unknown, and Native American). The two census tract-level categories were Yost Index SES, which was divided into a Low-Middle SES category (“Lowest”, “Lower”, and “Middle” quintiles) and a High SES category (“High” and “Highest” quintiles), and Rurality-Urbanicity, with rurality defined as either having a Rural-Urban Community Area code of “Non-Urban” or a US Census Rurality-Urbanicity designation of either “Mostly Rural” or “All Rural”.

### Statistical analysis

Age-adjusted, multivariate logistic models were used to analyze the relationship between the four patient clinicodemographic factors on the individual-level (sex, race-ethnicity) and community-level (Yost Index SES and Rurality-Urbanicity) and their impacts on patient outcomes. Independent covariates were set as the following: sex (female as comparator, male as reference), race-ethnicity (non-White as comparator, non-Hispanic White as reference), Yost Index SES (Lowest-Middle SES as comparator, Higher-Highest SES as reference), and Rurality-Urbanicity (“Rural”-RUCA designation and “Mostly Rural” and “All Rural”-URIC designations as comparator, “Urban”-RUCA and “Mostly Urban” and “All Urban”-URIC designations as reference).

Using these age-adjusted, multilevel models, logistic regressions were used to analyze treatment outcomes assessed that included dependent outcomes of whether patients underwent primary site surgery and whether they experienced delays-in-treatment (3 or more months from primary tumor diagnosis to initial interventional treatment when indicated). These age-adjusted, multivariate models were also utilized for cox proportional-hazards analyses and affiliated log-rank significance-testing to evaluate multi-level SDoH impact on all-cause mortality/overall survival. In addition, supplemental interaction-moderation logistic regresssion and Cox-proportional hazards models were performed utilizing these covariates and additional interaction terms of Yost Index*Sex and Yost Index*Minority Race/Ethnicity.

Chi-squared tests were conducted to investigate the differences between “High” (classified from “Higher” and “Highest”-Yost Index score) and Low-Middle Yost Index SES across clinicodemographic characteristics.

Two-sided p-values were used for all analyses. The alpha-criterion was set to 0.05. R-4.2.3 was used to perform statistical analyses.

## Results

44,514 patients diagnosed with pituitary adenomas were identified and analyzed from the SEER database. The most represented characteristics were females (*n* = 24,550, 55.2%), 20–44 years of age (*n* = 16,571, 37.2%), and white race (*n* = 23,261, 52.3%). Lower (*n* = 21,309, 48%) and higher-SES (*n* = 23,205, 52%) categories based on composite, community-level Yost Index measures were well-represented (Table 1).

**Table 1:** Clinicodemographic characteristics by yost-index-SES score

		Yost Socioeconomic Index Score		
Characteristic	*N*	**High SES**, *N* = 23,205 (52%)	**Low-Middle SES**, *N* = 21,309 (48%)	*p*-value
**Age**	44,514			0.183
20–44 years		8,581 (37%)	7,990 (37%)	
45–64 years		8,666 (37%)	7,838 (37%)	
65–84 years		5,417 (23%)	5,034 (24%)	
85 + years		541 (2.3%)	447 (2.1%)	
**Sex**	44,514			< 0.001
Female		12,419 (54%)	12,131 (57%)	
Male		10,786 (46%)	9,178 (43%)	
**Race**	44,514			< 0.001
White		14,189 (61%)	9,072 (43%)	
Hispanic		3,262 (14%)	5,311 (25%)	
Black		2,410 (10%)	5,511 (26%)	
Asian or Pacific Islander		2,693 (12%)	1,073 (5.0%)	
Unknown		431 (1.9%)	204 (1.0%)	
Native American		220 (0.9%)	138 (0.6%)	
**Rural-Urban Commuting Area Code**	42,778			< 0.001
Rural		511 (2.4%)	3,152 (15%)	
Urban		20,958 (98%)	18,157 (85%)	
**US Census Rural-Urbanicity**	42,778			< 0.001
All Rural		336 (1.6%)	1,382 (6.5%)	
All Urban		16,031 (75%)	14,839 (70%)	
Mostly Rural		830 (3.9%)	1,448 (6.8%)	
Mostly Urban		4,272 (20%)	3,640 (17%)	
**Primary Surgery Performed**	44,097			0.989
No Surgery		12,539 (54%)	11,472 (54%)	
Surgery		10,487 (46%)	9,599 (46%)	
**Months from Diagnosis to Initial Treatment**	31,275			< 0.001
< 1 month		5,227 (32%)	4,814 (32%)	
1–3 months		5,697 (35%)	4,721 (32%)	
3 months or more		5,442 (33%)	5,374 (36%)	
**3-year All-cause Mortality**	31,170			< 0.001
Alive, >3yr		15,100 (93%)	13,565 (90%)	
Dead, =<3yr		1,067 (6.6%)	1,438 (9.6%)	
**5-year All-cause Mortality**	24,318			< 0.001
Alive, >5yr		11,157 (89%)	9,812 (84%)	
Dead, =<5yr		1,449 (11%)	1,900 (16%)	
**Vital Status on Last Follow-up**	44,514			< 0.001
Alive		21,019 (91%)	18,573 (87%)	
Dead		2,186 (9.4%)	2,736 (13%)	

Across sociodemographic characteristics, chi-squared analyses showcased significant differences across lower and higher-Yost-Index-SES categories based on sex (*p* < 0.001), race (*p* < 0.001), Rural-Urban Commuting Area Code and US Census Rural-Urbanicity (*p* < 0.001). Other clinical characteristic differences were also observed (Table 1).

### Mortality

Through age-adjusted multivariate models, overall risk for mortality was markedly increased with community-level factors of lower Yost-Index SES (HR 1.43, 95%CI 1.35–1.52) and nearly with increasing rurality (1.09, 1.00-1.20) while individual-level minority status did not. Inversely, markedly decreased risk was observed with Female sex (0.81, 0.76–0.86) (Table 2). Across interaction models, no significant conditional differences were observed between Yost Index and individual-level characteristics of minority status and sex (Supplement Table 1).

**Table 2:** Age-adjusted, multilevel cox-proportional hazards model of survival differences

Disease Classification	Characteristic	HR	95% CI	*p*-value
Pituitary Adenomas, NOS	Sex - Female	0.81	0.76, 0.86	< 0.001
	Minority Race/Ethnicity	1.02	0.96, 1.09	0.439
	Yost Index - Lower Socioeconomic Status	1.43	1.35, 1.52	< 0.001
	Rurality	1.09	1.00, 1.20	0.059

### Treatment

Using similar age-adjusted, multivariate models, receipt of indicated surgery was markedly and independently increased with individual-level factors of minority race-ethnicity (OR 1.18, 95%CI 1.14–1.23) and community-level factors of increasing rurality (1.08, 1.01–1.16) while lower community-level Yost-Index SES did not. Surgical intervention was markedly decreased was observed with Female sex (0.61, 0.58–0.63) (Table 3). No significant conditional associations were observed among Yost-Index SES and individual-level covariates of minority status and sex (Supplement Table 2).

**Table 3:** Age-adjusted, multilevel logistic regression model of indicated surgery receipt

Disease Classification	Characteristic	OR	95% CI	*p*-value
Pituitary Adenomas, NOS	Sex - Female	0.61	0.58, 0.63	< 0.001
	Minority Race/Ethnicity	1.18	1.14, 1.23	< 0.001
	Yost Index - Lower Socioeconomic Status	0.98	0.94, 1.02	0.241
	Rurality	1.08	1.01, 1.16	0.030

Regarding delays in the initiation of indicated, interventional treatment, both community-level factors of lower Yost Index-SES (OR 1.14, 95%CI 1.08–1.20)) and individual-level female sex (1.24, 1.18–1.30) markedly and independently increased rates of delays whereas residing in rural communities and being of minority race/ethnicity did not (Table 4). These findings were corroborated among interaction models, where lower Yost-Index SES conditionally increased odds of treatment initiation delay among patients of female sex (interaction-OR 1.16, 1.05–1.28) (Supplement Table 3).

**Table 4:** Age-adjusted, multilevel logistic regression model of interventional treatment delays

Disease Classification	Characteristic	OR	95% CI	*p*-value
Pituitary Adenomas, NOS	Sex - Female	1.24	1.18, 1.30	< 0.001
	Minority Race/Ethnicity	0.98	0.93, 1.03	0.377
	Yost Index - Lower Socioeconomic Status	1.14	1.08, 1.20	< 0.001
	Rurality	0.95	0.88, 1.04	0.295

## Discussion

Through utilizing a nationally representative, specialized pituitary adenoma patient cohort, alongside robust multilevel modeling approaches, this investigation comprehensively assesses a variety of SDoH factors for national disparities in the care and prognosis of pituitary adenomas. Using multilevel, age-adjusted modeling with the Yost Index, alongside other community-level and individual-level SDoH-factors, this study showcased how community-level factors quantifiably show stronger associations with overall mortality for pituitary adenoma patients while showing significant but comparable associative effects with treatment initiation and receipt to individual-level factors. By addressing the shortcomings of prior studies limited by regional scope of patients, variable selection in modeling, geographic specificity of area-level characteristics, this multilevel analysis represents a broad investigation into how specific SDoH-factors associate with pituitary adenoma care and prognosis disparities in the US.

Increased all-cause mortality risk was observed among pituitary adenoma patients with worse community-level Yost Index-SES scores areas. While prior studies have identified similar associations between limited aspects of individual-level SES (i.e. income), smaller subsets of pituitary adenoma patients, or less-specific area-level measures of SES with pituitary adenoma patient-overall survival [14], this present study leverages a composite, community-level measure to account for surrounding resources and other confounders beyond these prior individual-level investigations. Furthermore, our present utilization of the Yost Index incorporates its specificity on the level of US census tracts across a nationally-spanning population of pituitary adenoma patients. In doing so, substantially higher risk magnitudes were observed compared to previous studies by accounting for community-level factors. Although such patterns may be partly attributed to factors directly related to pituitary adenomas themselves, such as our observations related to delayed treatment initiation and decreased surgical receipt, prior investigations have ascertained that other causes outside of pituitary adenoma tumor burden itself, including aspects related to subsequent comorbidities or overlapping chronic conditions, may be the drivers of these composite-SES-level disparities [17, 18]. Notably, despite our dataset incorporating several high-fidelity sociodemographic characteristics, clinical details pertaining to quality-of-life metrics, related (chronic) comorbidities, and disease-specific/or other sources of causes of mortality among our pituitary adenoma patient cohort are not well-delineated within our present study. These shortcomings present a future investigative need to incorporate datasets with longitudinal data or a wider extent of comorbid factors beyond the outcomes described here. Nevertheless, the consistency of our findings as well as theirexacerbated magnitudes of association compared to prior studies suggest that structural community level disparities may have some level of association with worsened prognosis of pituitary adenoma patients (at least partly) beyond observations of prior analyses.

Individual-level female sex was also independently associated with lower all-cause mortality/increased overall survival among pituitary adenoma patients. This finding aligns with prior literature where females with a pituitary adenoma were similarly found to have lower mortality rates compared to their male counterparts [16–19]. To better understand potential factors underlying this phenomenon, our results uniquely but paradoxically observed how decreased surgery receipt and increased interventional treatment delay odds were associated with individual-level female sex. As aforementioned, these downstream observations from diagnosis to treatment may partly contribute to observed disparities pertaining to female sex. However, prior research has purported that the survival advantage in females is unlikely to be explained solely by treatment patterns (as observed here) and may instead reflect underlying biological factors of sex that are omnipresent across overall medical diseases of inquiry regardless of etiology [20–22]. Thus, despite these concerning, detrimental trends of treatment receipt and delays within patients diagnosed with pituitary adenomas, they may not truly associate with survival differences. However, given the sparse literature and the associative nature of our findings, further mechanistic studies on potential biological and clinical causes behind this apparent survival benefit will need to be conducted in order to understand how individual-level sex confers independent, beneficial survival associations beyond our adjusted-for, multilevel factors.

Of note, longer interventional treatment delays were observed with worse community-level Yost-SES scores despite similar rates of surgery receipt between the groups. At first, these findings seem paradoxical when compared to the large body of head-neck tumors with SES-vulnerabilities driving increased treatment delay, which then confer lesser (curative) surgery receipt and eventual increased mortality [14, 18]. However, pituitary adenomas are different in their clinical management, which are managed conservatively until clinical symptoms, such as hormonal dysregulation or optic compression, are found [23]. Given this context, our results elucidate how treatment delays in a typically benign, slow-growing tumor type may not translate to poorer prognostic outcomes and eventual indicated surgical receipt [24].

In contrast, unlike community-level factors like Yost Index-SES, being of a minoritized-race-ethnicity on the individual-level (compared to being of a non-Hispanic white race-ethnicity) saw no independent significant differences in mortality or treatment delays. However, minoritized patients diagnosed with pituitary adenomas were significantly more likely to receive surgery. Prior studies principally focused on individual-level race-ethnicity in pituitary adenomas and other neuroendocrine tumors have denoted similar trends, with non-white patients often presenting with larger or more symptomatic tumors, which may contextually drive higher intervention rates [25, 26]. As aforementioned, considerations of the discordance between eventual surgical treatment receipt without significant differences in treatment delay nor mortality may be contextualized in the initial conservative management of pituitary adenomas. However, as our findings cannot confer causal inferences, future investigations should conduct prospective or qualitative approaches for understanding treatment patterns and symptomatic management among patients and providers of pituitary adenomas. Nevertheless, our multilevel analysis further validates prior results of assessing independent racial-ethnic surgery receipt trends by adjusting for a multitude of multilevel factors.

Interestingly, our findings also displayed how increasing rurality was associated with decreased surgical receipt and nearly with increased overall mortality. Although such findings have been indirectly assessed in prior literature, primarily in increased pituitary adenoma incidence among more-urban populations [12], direct quantitative relationships pertaining to urbanicity-rurality and outcomes have not been ascertained to date. Beyond measures of incidence, other indirectly related measures, including aspects of surgical provider geographical distribution, high-/low-surgical volume centers, and private vs. safety-net hospital access, have been assessed within the context of pituitary adenoma care receipt, active surveillance recommendations, and loss to follow-up trends [27–30]. However, as these prior investigations have only assessed these institutional-based characteristics from the lens of urban/metropolitan settings without consideration of a patient’s living circumstances, they do not fully represent the measures of urbanicity-rurality directly measured within our study, as facility traits may vary whether in an urban or rural setting. Although our findings are uniquely observed among pituitary adenoma patients within the US, they corroborate with the larger body of work among area-based SDoH-trend analyses in general head-neck and central nervous system tumor investigations, which observed how increased vulnerability in built infrastructure access and housing/transportation-status portended worse care outcomes across the board [7, 11, 31–34].

Among the strengths of this study, the use of a large, nationally representative cohort with a relevant chronology sampling of patients lends to this investigation’s generalizability. In addition, as a dataset specially authorized for highlighting census-level variables alongside individual-level characteristics, considerations of these multilevel factors allows for a more comprehensive modeling of outcomes, including its provision of the census-level, SES-composite measure of the Yost Index. However, this study also features several key limitations to be considered with its approach. As with any retrospective, population-level data, the inability to encompass all clinical and sociodemographic-relevant variables, including watchful waiting strategies as treatment, optic chiasm involvement, hormonal activity, and comorbidity status, among others, remains an important gap for not elucidating clinical contexts behind observed outcomes. In addition, its retrospective observational design presents inherent weaknesses for not enabling causal inferences. Furthermore, considerations of other community-level factors, beyond the current Yost-Index and rurality-urbanicity measures, are hindered due to the deidentified and HIPAA-compliant nature of the SEER dataset. In addition, limitations regarding discernment of perioperative outcomes were not available due to restrictions of the dataset utilized. Future investigations incorporating propriety or private datasets with a wider and more recent spectra of clinicodemographic variables and outcomes would be warranted to address proposed questions of mechanisms underlying these multilevel disparity associations.

This SDoH-based investigation in pituitary adenoma differences not only builds upon prior work regarding socioeconomic, sex, and racial-ethnic differences in mortality, treatment delays, and surgery receipt, but also contextualizes their interactive effects through jointly assessing a variety of individual and community-level variables. By simultaneously examining mortality, timing of interventional treatment initiation, and surgery receipt through this comprehensive lens, our findings highlight the multifaceted associations of individual and community-level SDoH factors with treatment and survival outcomes. These results not only validate prior observations of SDoH-derived disparities but also emphasize the importance of accounting for structural, community-level drivers of care delays and mortality. In doing so, more comprehensive foundations for prospective investigations and implementation efforts can strategically and objectively target resources towards the most pertinent SDoH-factors driving pituitary adenoma disparities.

## Supplementary Information

Below is the link to the electronic supplementary material.


Supplementary Material 1



Supplementary Material 2



Supplementary Material 3



Supplementary Material 4


## Data Availability

The datasets analyzed during the current study are available through the National Cancer Institute Surveillance, Epidemiology, and End Results (SEER) program ([https://seer.cancer.gov/](https://seer.cancer.gov)) and the Centers for Disease Control and Prevention Social Vulnerability Index repository ([https://www.atsdr.cdc.gov/place-health/php/svi/index.html](https://www.atsdr.cdc.gov/place-health/php/svi/index.html)).
