# Supplementary material for: Individual and community-level associations of social determinants with pituitary adenoma disparities in the US
Source: J Neurooncol. 2026 Aug 5;179(1):29. doi: 10.1007/s11060-026-05716-y (PMC13442616; doi:10.1007/s11060-026-05716-y)
Supplement: Supplementary file 1 [file 11060_2026_5716_MOESM1_ESM.docx]

| Disease Classification^1^ | **Characteristic** | **HR** | **95% CI** | **p-value** |
| --- | --- | --- | --- | --- |
| **Pituitary Adenomas, NOS** | **Sex - Female * Yost Index - Lower Socioeconomic Status** | 0.99 | 0.88, 1.12 | 0.897 |
|  | **Minority Race/Ethnicity * Yost Index - Lower Socioeconomic Status** | 1.12 | 0.99, 1.27 | 0.066 |
|  | | | | |
