# Supplementary material for: Individual and community-level associations of social determinants with pituitary adenoma disparities in the US
Source: J Neurooncol. 2026 Aug 5;179(1):29. doi: 10.1007/s11060-026-05716-y (PMC13442616; doi:10.1007/s11060-026-05716-y)
Supplement: Supplementary file 4 [file 11060_2026_5716_MOESM4_ESM.docx]

**Supplement Table 1. Age-Adjusted, Interaction-Multilevel Cox-Proportional Hazards Mode of Overall Survival Differences**

**Supplement Table 2. Age-Adjusted, Interaction-Multilevel Logistic Regression Model of Indicated Surgery Receipt**

**Supplement Table 3. Age-Adjusted, Interaction-Multilevel Logistic Regression Model of Interventional Treatment Delays**
